# Effect of educational interventions addressing blood groups and donation aspects on the knowledge and attitude of allied health sciences students

**DOI:** 10.12669/pjms.40.10.8803

**Published:** 2024-11

**Authors:** Nazish Saqlain, Hafsa Habib, Shazia Riaz, Sidra Hareem

**Affiliations:** 1 Nazish Saqlain, FCPS Associate Professor of Pathology, Department of Hematology & Transfusion Medicine, University of Child Health Sciences (UCHS), The Children’s Hospital Lahore, Pakistan; 2 Hafsa Habib, B.Sc Medical Laboratory Technologist, Department of Hematology & Transfusion Medicine, University of Child Health Sciences (UCHS), The Children’s Hospital Lahore, Pakistan; 3 Shazia Riaz Department of Pediatric Hematology/Oncology, University of Child Health Sciences (UCHS), The Children’s Hospital Lahore, Pakistan; 4 Sidra Hareem, FCPS Assistant Professor of Pathology, Department of Hematology & Transfusion Medicine, University of Child Health Sciences (UCHS), The Children’s Hospital Lahore, Pakistan

**Keywords:** Educational interventions, Blood groups, Blood donation, Students, Questionnaire

## Abstract

**Objective:**

To determine the effect of educational interventions on knowledge and approach of Allied Health Sciences Students regarding blood groups and blood donation.

**Methods:**

It was a cross-sectional study conducted at the department of Hematology and Transfusion Medicine, University of Child Health Sciences, The Children’s Hospital, Lahore from October 2022-January 2023. A self-designed questionnaire was used to assess the baseline knowledge of students followed by educational interventions in the form of small group discussions, video lectures and practical demonstrations. Post-interventions assessment was done using the same questionnaire 30 days later. Data obtained was analyzed using SPSS-23 software. A p-Value <0.05 was considered significant. The reliability of the questionnaire was checked after the pilot study by using the SPSS-23 software and calculated Cronbach’s Alpha was 0.82.

**Results:**

Of the 99 participants, there were 13 males and 86 females with mean age of 21.41±1.478 years. Pre-interventions, majority participants had moderate knowledge (81.8%). Medical Lab technologists showed better baseline knowledge than the rest. Post-interventions, the level of knowledge among students increased to 94.9%. Pre-interventions, knowledge about blood groups and donation ranged from 14.1-79% and 25.5-77.8% correct responses respectively while post-interventions it went up to 92-100% for both aspects. The willingness to donate blood in future raised from 46.4% to 92.9%.

**Conclusions:**

Educational interventions were effective in improving the knowledge of Allied Health Sciences students about blood groups and blood donation. The enlightenment strategies positively influenced students towards the active participation in blood donation campaigns.

## INTRODUCTION

Blood donation is an essential aspect of healthcare as it supplies hospitals and healthcare facilities with a steady and safe blood inventory. Blood donation drives raise awareness about the importance of donating blood, encouraging individuals to contribute to their communities.^1^ On average 118.5 million blood donations are collected around the world each year with only 38% donation rate in the developing countries.^2^ Young adults of any population make major pool of healthy blood donors. Luckily, we as nation consist of large number of young people but unfortunately, we lag behind in voluntary blood donation. Approximately, annual blood collection is three million units in Pakistan.^3^

Shortage of blood, suboptimal donor care with poor retention, high number of directed blood donations, inadequate components preparation, fragmented transfusion traceability & reactions reporting are some of the challenges faced by transfusion services in our country.^3^,^4^ Hemovigilance ensures donor as well as recipient’s safety. The first step in achieving this goal is the recruitment and retention of healthy voluntary blood donors.^5^,^6^ Students particularly of medical and paramedical specialties can become enthusiastic voluntary blood donors and can act as best advocates to encourage and raise awareness in the society about blood donation. Educational interventions are of paramount importance in equipping students with knowledge and skills related to transfusion and donation aspects.^7^

The University of Child Health Sciences, Lahore, currently educates and provide training to Allied health sciences students, including medical laboratory (MLT), Dental (DT) and Operation theater technologists (OTT) and Doctors of Physical Therapy (DPT) who become directly involved in patient care and transfusion-related procedures. It is vital for these students to have a comprehensive understanding of blood groups and donation aspects to ensure patient safety. The study was focused to find the baseline knowledge of the students, let them participate in the educational activity and then determine its impact. The objective of the study was to determine the effect of educational interventions on the knowledge of Allied Health Sciences Students regarding blood groups and blood donation and to find their approach towards voluntary blood donation.

## METHODS

It was a cross-sectional study, conducted at the department of Hematology and Transfusion Medicine, University of Child Health Sciences, Lahore from October 2022-January 2023.

### Ethical Approval:

The study was approved by the Institutional Review Board (1209/SAHS dated 11/08/2022).

In this study, 99 students from four disciplines, that is MLT, DT, OTT and DPT studying during research period from 2^nd^ to 4^th^ year classes were included through consecutive sampling. Informed consent was taken from all the participants and the confidentiality was maintained at each level of the response in the study. The students who did not give consent or those who could not attend all educational sessions or had incomplete post-intervention proforma were excluded.

Data was collected on a self-designed questionnaire, the reliability of which was checked after the pilot study and calculated Cronbach’s Alpha was 0.82. The questionnaire (Appendix) consisted of four sections. Section A, comprised of sociodemographic details, section B consisted of 14 questions (Q1-14) that assessed participants’ knowledge of blood groups, section C consisted of 15 questions (Q15-29) that assessed knowledge about blood donation and section D comprised of two questions (Q30-31) to see the approach of students towards future blood donation. The questionnaires were filled by students who gave written informed consent to participate in the study. This was followed by education in the form of a small group discussions, video lectures, and practical, hands-on session. The interventions applied were same for all the students. One month after the educational interventions, the same questionnaire was administered to the same groups of students in order to assess their effect.

For the scoring of variables, a score of one was awarded for correct answers and zero for wrong or unanswered question, giving a range of 0-31. Scores were converted to percentages and getting all the correct answers score of 100% was given while getting none of the questions correctly were scored as 0%. The scores were graded as poor knowledge (scores 0%-49.9%), fair knowledge (scores between 50% and 79.9%), and good knowledge (scores 80%-100%).

### Data analysis:

Data was analyzed by SPSS version 23.0. Continuous variable such as age is expressed in the form of Mean± SD, whereas categorical variables are in the form of frequencies and percentages. Bar chart was used to display the frequencies. A chi square test was used to compare the results of pre- and post- interventions with p-Value ≤0.05 was considered significant.

## RESULTS

Among 99 students, 60(60.6%) were from MLT, 16 (16.2) were from DPT, 15 (15.2) from DT and 8 (8.1) from OTT disciplines. They comprised of 13 male and 86 female students with a ratio of 1:6.6. The mean age was 21.41±1.478 years. Only 10.1% participants had previous experience of blood donation, (Table-I).

**Table-I:** Sociodemographic Characteristics of Participants.

Characteristics	Frequency (%)
***Gender***	
Males	13(13.1)
Females	86(86.9)
***Age(years)***	
18-20	27(27.2)
20-22	48(48.4)
22-24	25(25.2)
Mean±SD	21.41±1.478
***Educational Disciplines***	
MLT	60(60.6)
DPT	16(16.2)
DT	15(15.2)
OTT	8(8.1)
***Previous Experience of Blood donation***	
Yes	10(10.1)
No	89(89.9)

Total	99(100)

Grades of knowledge before and after educational interventions among different disciplines are shown in Table-II,. Medical lab technologists showed better baseline knowledge about blood groups and donation. Post-interventions, >80% score rate achieved among 75-100% students of different disciplines.

**Table-II:** Pre- and Post-Intervention Blood Groups and Blood Donation Knowledge grading among different Allied Health Sciences disciplines.

Disciplines	Educational Interventions	Knowledge level		

		Poor knowledge (<50% score)	Fair Knowledge (50-80%)	Good knowledge (>80%)
MLT	Pre	0(0)	52(86.6)	4(6.7)
	Post	0(0)	0(0)	60(100)
DPT	Pre	2(13)	13(81)	1(6.2)
	post	0(0)	0(0)	16(100)
DT	Pre	5(33.3)	10(66.6)	0(0)
	Post	0(0)	3(20)	12(80)
OTT	Pre	2(25)	6(75)	0(0)
	Post	0(0)	2(25)	6(75)

The knowledge about blood groups before educational interventions showed < 50% correct responses to Q8, Q9, Q10 and Q13 were obtained. The questions addressed different blood group systems apart from ABO, nature of anti-A and anti-B antibodies, forward and reverse blood grouping technique and Weak D antigen detection respectively. Post- educational interventions, the frequency of correct responses ranged from 92-100%. Educational interventions showed positive significance in terms of improving knowledge for 13 out of 14 questions, Table-III.

**Table-III:** Pre- and Post- Educational Interventions Correct and Incorrect Responses about Blood Groups.

		Pre-Interventions N (%)		Post-Interventions N (%)		(X^2^)P-value

		Responses				

		Correct	Incorrect	Correct	Incorrect	
Q1	Which blood group is the Universal Recipient for RBCs conc. transfusion?	61(61.6)	38(38.3)	97(98)	2(2.0)	0.001
Q2	Which blood type is considered as a universal whole blood donor?	57(57.6)	42(42.4)	94(95)	5(5.0)	(46.06) 0.001
Q3	What antigens are present on RBCs in O blood group individuals?	64(64.6)	35(35.3)	97(98)	2(2.0)	0.001
Q4	What antibodies are present in serum of O blood group individuals?	59(59.5)	40(40.4)	98(99)	1(1.0)	0.001
Q5	Which blood group is the first choice in terms of emergency plasma transfusion?	57(57.7)	42(42.4)	94(95)	5(5.0)	0.001
Q6	What are methods of blood group determination?	52(52.5)	47(47.4)	99(100)	0(0)	0.001
Q7	What is the interpretation of blood groups for given results?	79(79.7)	20(20.2)	99(100)	0(0)	(8.756) 0.045
Q8	What are different Blood group systems other than ABO?	33(33.3)	66(66.7)	99(100)	0(0)	0.001
Q9	What is the nature of anti-A and anti-B antibodies?	14(14.1)	85(85.9)	91(92)	8(8.0)	0.001
Q10	Can you interpret results of Forward and reverse blood grouping?	30(30.3)	69(69.6)	93(94)	6(6.0)	0.001
Q11	What are Rh antibodies in nature?	46(46.4)	53(53.5)	95(96)	4(4.0)	0.001
Q12	Which clinical conditions are associated with Rh antibodies?	63(63.6)	36(36.3)	99(100)	0(0)	0.001
Q13	Do you know about Weak D antigen?	29(29.2)	70(70.7)	95(96)	4(4.0)	0.001
Q14	Do you know the blood group associated with resistance to P. vivax malaria?	45(45.5)	54(54.5)	99(100)	0(0)	0.001

Pre-educational interventions, the responses to questions regarding annual blood donation frequency, suitable age for blood donor, time required per donation, blood donation during fasting and storage conditions for blood components were less than 50%. Post- educational interventions, the frequency of correct responses for different questions ranged from 91-100%. Significant impact of these interventions was found in getting correct answers to 11/15 questions, Table-IV.

**Table-IV:** Pre- and Post- Educational Interventions Correct and Incorrect Responses about Blood Donation.

	Questions	Pre-Interventions N (%)		Post-Interventions N (%)		(X^2^) P-value

		Responses				

		Correct	Incorrect	Correct	Incorrect	
Q15	What is the volume of donated blood?	57(57.6)	42(42.4)	96(97)	3(3.0)	0.007
Q16	How many times a donor can donate blood annually?	39(39.4)	60(60.6)	94(95)	5(5.0)	0.002
Q17	What is the suitable age of a blood donor?	25(25.2)	74(74.7)	90(91)	9(9.0)	0.034
Q18	What is the minimum weight of a donor?	57(57.6)	42(42.4)	99(100)	0(0)	0.001
Q19	Which Transfusion transmissible infections (TTI) are mandatory to be screened in allogenic blood units in Pakistan?	71(71.7)	28(28.2)	98(99)	1(1.0)	(13.08) 0.002
Q20	What is the duration of blood donation process?	26(26.2)	73(73.7)	95(96)	4(4.0)	0.001
Q21	Can one donate blood while fasting?	35(35.4)	64(64.6)	98(99)	1(1.0)	0.001
Q22	Can a person develop anemia after blood donation?	56(56.6)	43(43.4)	99(100)	0(0)	0.001
Q23	Can blood be separated into components?	77(77.8)	22(22.2)	99(100)	0(0)	(13.27) 0.001
Q24	Can you tell names of different blood products?	77(77.8)	22(22.2)	99(100)	0(0)	(13.27) 0.001
Q25	Do you know that one unit of donated blood can be used for more than one patient?	66(66.7)	33(33.3)	99(100)	0(0)	0.001
Q26	Do you think you can do your routine job next day after donation?	77(77.8)	22(22.2)	99(100)	0(0)	(13.27) 0.001
Q27	Do you know about directed, replacement and voluntary blood donors?	83(83.8)	16(16.2)	99(100)	0(0)	
Q28	Can you donate after COVID vaccination/resolved infection?	59(59.6)	40(40.4)	98(99)	1(1.0)	
Q29	Do you know that blood components can be stored?	47(47.5)	52(52.5)	92(93)	7(7.0)	0.001

**Fig.1:**
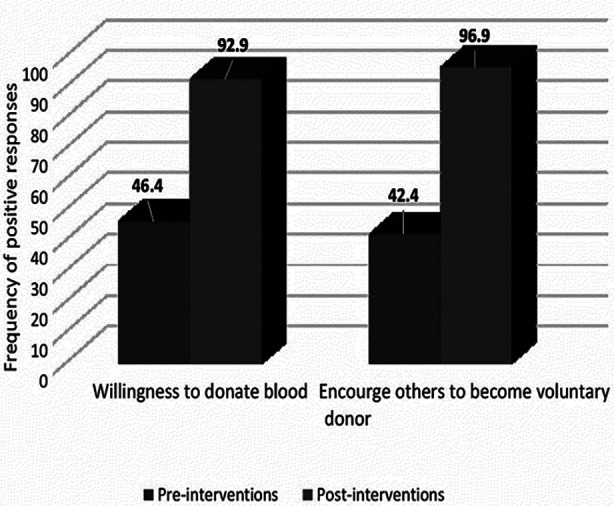
Pre- and Post- educational interventions positive responses about voluntary blood donation.

After the educational interventions, the willingness to donate blood in future on voluntary basis raised from 46.4% to 92.9% among the participants. While 96.9% said that they would encourage their friends, family and colleagues to participate actively in blood donation campaigns after educational sessions, Fig.1.

## DISCUSSION

The rapidly evolving healthcare technologies have so far failed to provide an alternative non-human method for supplying blood and blood products. In a developing country like ours, lack of knowledge, blood donor recruitment and retention plan and various myths have resulted in limited number of voluntary donations.^4^,^8^ This challenge can be tackled by adopting such strategies that can overcome the misconceptions and also motivate the public to donate at an early age so that they become lifelong voluntary donors. Medical and Paramedical students can play a pivotal role in this regard. Our study focused on such educational interventions involving the Allied Health Sciences Students.

The study revealed that the most of the participants were 20-22 years old. Majority of the students had no previous blood donation experience showing non-participation in blood donation drives at community level. Infrequent history of donation among medical students have also been reported by Aslami et al (10%), Devi et al (14%) and Nwogoh et al (22%).^9^-^11^ Samira AW et al found that majority of medical students had not donated blood despite good knowledge of blood donation.^12^ We found that educational interventions significantly improved students’ knowledge about blood donation and blood groups. At baseline, 81.8% of the participants had fair knowledge while post intervention, 94.9% were in the good knowledge category. The findings are similar to the previous studies.^13^,^14^

Before educational interventions, correct responses for questions related to blood group systems ranged from 14-79%. The items with poor response were about minor blood group systems, Weak D and forward and reverse blood grouping technique. Students’ acquaintance of blood groups improved after educational interventions. The findings are concordant with previous studies in terms of few questions.^9^,^13^ Most of the studies have only focused on common blood group systems or students’ own ABO and Rh blood group.^13^,^15^,^16^ Regarding baseline knowledge about blood donation, correct responses to five out of fifteen questions were particularly low (<50%).

After the educational interventions, correct response rate increased significantly for majority of the items. Ugwu NI et al had shown similar effect of educational strategies in increasing knowledge of students regarding blood donation.^13^ Gupta et al. studied 135 paramedical students and revealed the knowledge gap about blood donation. It was also pointed out that students who had donated blood previously had better knowledge of the process.^17^ Sabu KM have reported good level of knowledge among allied health sciences students (53.1%).^18^ Similar findings are revealed by other studies including medical students.^19^

However, a previous study showed poor knowledge of blood donation among medical students which could have been contributed to the selection of undergraduate students in the early years of medical education.^9^ Before educational interventions, about half of the students were willing to donate voluntarily and want themselves to be registered as a voluntary blood donor. Similar number showed commitment to encourage their family and friends to participate in blood donation camps. Martínez-Santos AE and colleagues have revealed low knowledge scores among medical students and have stressed on developing specific strategies in curriculum to encourage students to become regular blood donors.^20^ We found a significant change in the approach of students towards voluntary blood donation after educational sessions. Similar findings are reported by previous studies.^13^,^19^,^21^

The basic goal of blood donor education is to promote knowledge, attitudinal change, and to educate the donors about self-selection and self-exclusion. This study has revealed the knowledge gap among paramedical students. However, at the same time, this study has shown potential change in their knowledge and attitude with the help of specific educational interventions. Donor education relieves fears and strengthen public confidence in safe blood supply.^22^ This can be facilitated through simple but clear messages to the target audience, particularly young potential donors, starting the efforts with healthcare providers.

### Limitations:

The study has some limitations including selection bias in terms of gender representation. Implementation of change in students’ attitude towards voluntary blood donation could not be seen as the study was time bound. However, future studies can focus on educational sessions and measurement of their effects through candidates’ participation in blood donation camps.

## CONCLUSION

Educational interventions are effective in improving the knowledge of Allied Health Sciences students about blood groups and blood donation. The attitude toward voluntary blood donation can be improved with educational interventions. Therefore, there is a need for frequent educational sessions among potential blood donors to ensure a sustained pool of voluntary blood donors particularly in our part of the world.

### Authors’ Contribution:

**NS:** Designed the research, contributed in manuscript writing and gave final approval of the version to be published.

**HH:** Designed Questionnaire and conducted survey, Collected & Interpreted data, drafted and compiled the initial version of manuscript.

**SR:** Collected data, drafted manuscript, critically reviewed the final version.

**SH:** Conducted survey, collected and analyzed data, wrote and revised manuscript.

All authors are equally responsible and accountable for ensuring the accuracy and integrity of any part of this work.
